# Nitrogen-Doped Oxygenated Molybdenum Phosphide as an Efficient Electrocatalyst for Hydrogen Evolution in Alkaline Media

**DOI:** 10.3389/fchem.2020.00733

**Published:** 2020-08-28

**Authors:** Muhammad Waqas Khan, Suraj Loomba, Rashad Ali, Md Mohiuddin, Ahmed Alluqmani, Farjana Haque, Yongkun Liu, Rizwan Ur Rehman Sagar, Ali Zavabeti, Turki Alkathiri, Babar Shabbir, Xian Jian, Jian Zhen Ou, Asif Mahmood, Nasir Mahmood

**Affiliations:** ^1^School of Engineering, RMIT University, Melbourne, VIC, Australia; ^2^Applied Porous Materials Unit, Commonwealth Scientific and Industrial Research Organisation (CSIRO), Clayton, VIC, Australia; ^3^School of Materials and Energy, University of Electronic Science and Technology of China, Chengdu, China; ^4^College of Textile Science and Engineering (International Institute of Silk), Zhejiang Sci-Tech University, Hangzhou, China; ^5^School of Materials Science and Engineering, Jiangxi University of Science and Technology, Ganzhou, China; ^6^Department of Chemical Engineering, The University of Melbourne, Parkville, VIC, Australia; ^7^School of Engineering, Albaha University, Al Bahah, Saudi Arabia; ^8^Department of Materials Science and Engineering, ARC Centre of Excellence in Exciton Science, Monash University, Clayton, VIC, Australia; ^9^National Engineering Researching Centre of Electromagnetic Radiation Control Materials, State Key Laboratory of Electronic Thin Films and Integrated Devices, University of Electronic Science and Technology of China, Chengdu, China; ^10^School of Chemical and Biomolecular Engineering, The University of Sydney, Sydney, NSW, Australia

**Keywords:** molybdenum phosphide, hydrogen evolution reaction, alkaline electrolyte, water-splitting, catalysts

## Abstract

Phosphides of transition metals (TMPs) are a developing class of materials for hydrogen evolution reaction (HER) as an alternative to expensive noble metals to produce clean energy. Herein, the nitrogen-doped molybdenum oxide (MoO_x_) is developed via a facile and simple hydrothermal method, followed by annealing in the N_2_ atmosphere and phosphorization to form a nitrogen-doped oxygenated molybdenum phosphide (N-MoP) sphere-shaped structure. The developed N-doped phosphide structure depicts enhanced HER activity by reaching a current density of 10 mA cm^−2^ at a very low overpotential of only 87 mV, which is much better than annealed nitrogen-doped molybdenum oxide (A-MoO_x_) 138 mV in alkaline medium. N-MoP is a highly efficient electrocatalyst for HER attributed to a more exposed surface, large electrode/electrolyte interface and appropriate binding energies for reactants. This study extends the opportunity of developing nitrogen-doped TMPs, which can display exceptional properties as compared to their oxides.

## Introduction

Excessive use of fossil fuels and other non-renewable energy sources has led to their fast depletion and the omnipresent issue of environmental pollution (Jiao et al., 2015; Yuan et al., 2016; Li et al., 2020). It is urgent to find out the alternative sources of energy that have the potential to replace the swiftly exhausting fossil fuels. Hydrogen, due to its recyclability and high energy density, has emerged as a clean energy source (Mahmood et al., 2018; Haque et al., 2019; Ren Q. et al., 2019; Surendran et al., 2019b; Aslam et al., 2020). The electrochemical water splitting via hydrogen evolution reaction (HER) is expected to provide a favorable pathway for inexpensive H_2_ generation, which can be used as an alternative fuel to fossil fuels (Turner, 2004; Zou and Zhang, 2015; Surendran et al., 2018, Surendran et al., 2019a). Noble metals such as platinum (Pt/C) perform exceptionally well when used as an electrocatalyst for HER in acidic media (Xiao et al., 2014); however, its wide-scale application is hindered due to high cost and scarcity in the earth's crust (Xiao et al., 2014; Zhou et al., 2017; Li et al., 2020). Therefore, the focus has been shifted toward finding the electrocatalysts which are earth abundant and inexpensive and possessed a similar or better performance than precious metals. Recently, transition-metal alloys, nitrides, sulfides, oxides, selenides, phosphides, borides, and carbides have been studied (Fosdick et al., 2014; Yuan et al., 2016; Zhang et al., 2017; Zhao et al., 2018; Lian et al., 2019; Zou et al., 2020). Out of these materials, transition metal phosphides (TMPs) have shown superior activity and stability (Ren Q. et al., 2019; Zou et al., 2020). Another challenge associated with electrocatalysts is the type of electrolyte being used during the reaction. Particularly, the highly corrosive nature of acidic media has increased the cost of setting up the system, producing safety concerns, and instability (Subbaraman et al., 2011). Thus, it is required to search for an effective electrocatalyst that can perform well in the basic media and have low energy requirements. In recent years, molybdenum-based compounds have emerged as potential candidates for HER (Li et al., 2018; Yao et al., 2018; An et al., 2019). Molybdenum base compounds showed better performance in the acidic medium as compared to the alkaline medium (Haque et al., 2019). For sustainable development of hydrogen as cleaner energy, HER in the alkaline medium is a point of focus (Mahmood et al., 2018). Initially, molybdenum sulfide was prepared to be applied as an electrocatalyst, but high overpotential requirement and poor stability in the alkaline medium have limited its long-term use (Anjum et al., 2018). Various other Mo-based compounds including carbides, borides, and nitrides were studied in the alkaline medium, but their use was hindered by poor stability and higher overpotentials (Haque et al., 2019). To meet up the challenges of developing an electrocatalyst with good performance and stability, the oxides of molybdenum are a good solution because at room temperature one of the oxides, i.e., molybdenum trioxide, is chemically inert (Datta et al., 2017). However, when it comes to long-term stability tests, these trioxides do not maintain a consistent performance which paved the way for MoP as an electrocatalyst (Xiao et al., 2014; Haque et al., 2019). Moreover, nitrogen doping helps in improving electrocatalytic performance by creating positively charged sites (Yuan et al., 2016; Sim et al., 2020). Experiments carried out by Xing et al. (2014) resulted in the development of the closely linked networked structure of MoP nanoparticles, which showed the best HER performance with an overpotential of 125 mV to achieve a current density of 10 mA cm^−2^. However, there is a considerable gap in understanding the HER activity on N-doped MoP. Therefore, there is a need to develop some nitrogen-doped MoP-based novel structures for an enhanced performance and stability in the alkaline medium. Previously, N-doped MoO_x_ by crystal phase transition was reported to have efficient and stable HER activity (Haque et al., 2019). Here, we present that the thermal conversion of nitrogen-doped MoO_x_ into phosphide can further enhance the HER catalytic activities in the alkaline medium. The nitrogen-doped molybdenum phosphide (N-MoP) is synthesized using a facile and simple hydrothermal method followed by annealing and phosphorization. The improved performance is due to the nitrogen doping along with thermal phosphorization of MoO_x_, which increases the active sites for electrocatalysis. N-MoP is tested in an alkaline medium in terms of overpotential, durability, stability, and electrochemical surface area for HER. This study extends the opportunity of developing nitrogen-doped TMPs, which can display exceptional properties as compared to their oxides.

## Experimental Section

### Materials

Hydrogen peroxide (30% weight) was used along with 99.9% pure Mo powder and 99% pure hexamethylenetetramine (HMTA) purchased from Sigma-Aldrich. Sodium hypophosphite (NaH_2_PO_2_) (98–101% weight, Sigma-Aldrich), polytetrafluoroethylene (PTFE) (60% weight, Sigma-Aldrich), potassium hydroxide (KOH, 85–90% weight), and Ni foam (99.9% purity, MTI Corporation). All chemicals were used without any further purification.

### Synthesis of Nitrogen-Doped Oxygenated Molybdenum Phosphide

The N-doped MoO_x_ is prepared as reported by Haque et al. (2019); 6 mL of hydrogen peroxide (30% weight) was added in 500 mg of Mo powder in a 20-mL glass vial to form peroxomolybdic solution. In another 20-mL glass vial, 350 mg of HMTA was dissolved in 20 mL of DI water. The HMTA dissolved in DI water was then slowly added into the peroxomolybdic solution and left for magnetic stirring for 30 min. The prepared solution was transferred into the Teflon-lined stainless-steel autoclave. The hydrothermal reaction was then carried out for 24 h at a temperature of 200°C. After experiment completion and cooling at room temperature, the sample obtained was washed with DI water and dried at room temperature, followed by annealing in a tube furnace under an N_2_ environment at a temperature of 350°C for 2 h at 3°C per min to get annealed MoO_x_ (A-MoO_x_). The ramp rate in the annealing process was 5°C min^−1^. Annealing was done to remove adsorbed water and oxygen from the sample as much as possible so that it would not interfere during the phosphorization. 25 mg of annealed sample along with 750 mg sodium hypophosphite was put in a ceramic boat for thermal conversion to phosphide. The ceramic boat was positioned at the center of the tube furnace. The reaction was then carried out at 400°C with a ramping rate of 3°C per min for 3 h under an N_2_ environment to obtain N-MoP.

### Material Characterizations

A FEI Quanta 200 scanning electron microscope (SEM) with an attached energy-dispersive X-ray spectrometer (EDS) was used to check the morphologies and to do the elemental analysis of the samples. The crystal structure of the sample was obtained through X-ray diffraction (XRD) analysis using a Bruker D4 endeavor. A thermo-scientific K-alpha system was used to conduct high-resolution X-ray photoelectron spectroscopy (XPS). The machine consisted of an Al Kα monochromated X-ray source through which the samples were scanned. The sample scanning was done with a dwell time of 50 ms^−1^ and pass energy of 50 eV. Electrochemical measurements were conducted using a CHI 760D electrochemical workstation (CH instruments).

### Electrochemical Measurements

Electrochemical measurements were conducted in 0.1 M KOH solution using a typical three-electrode setup. Ni foam coated with the sample was used as the working electrode, a graphite rod was used as the counter electrode, and Hg/HgO (in 1 M KCl aqueous solution) was used as the reference electrode. The Ni foam was properly cleaned with ethanol and DI water before coating samples on it. The reference electrode was converted to RHE after calibration along with all the potentials that have been mentioned. The polarization curves in the paper were iR-corrected, which were obtained after performing linear sweep voltammetry at a scan rate of 5 mVs^−1^. The working electrode was prepared by mixing 5 mg of resultant compound in a carbon black solution following the addition of 5 μL of PTFE solution and sonicating it for 10 min. The carbon black solution was prepared by mixing 20 mg carbon in 20 mL IPA and DI water mixture (4:1) followed by sonication for 10 min. After this, 100 μL of the solution was drop-cast on the Ni foam with an area of 0.25 m^2^ to get 0.4 mg mass loading.

## Results and Discussion

**Figure 1:**
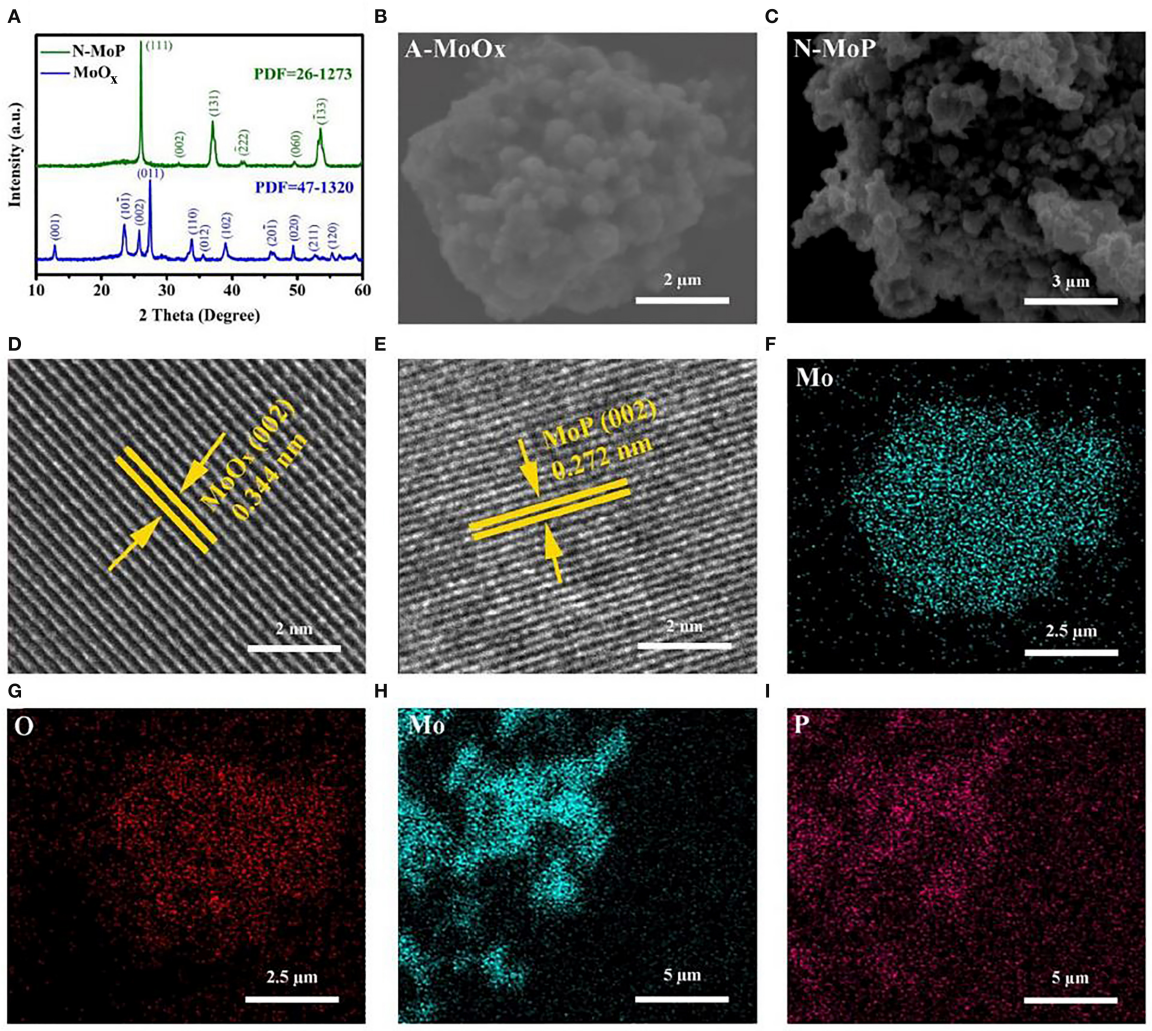
**(A)** XRD patterns of MoO_x_ and MoP, SEM images of **(B)** annealed MoO_x_, **(C)** N-MoP, HR-TEM images of **(D)** annealed MoO_x_, **(E)** N-MoP, EDS mapping of **(F)** Mo and **(G)** O elements in A-MoO_x_, EDS mapping of **(H)** Mo, and **(I)** P elements in the N-MoP sample.

The polycrystalline nitrogen-doped MoO_x_ was prepared using the hydrothermal method, annealed, and then thermally converted into N-MoP as described in the experimental section. The annealing process enhanced the crystallinity of A-MoO_x_, which shows enhanced performance due to the more positive charge sites, large electrode/electrolyte interface, and higher conductivity for electron/ions for HER in 0.1 M KOH. For the crystallographic study, the XRD analysis was performed for A-MoO_x_ and N-MoP; the XRD patterns are shown in Figure 1A. It is quite clear that the XRD patterns match well with the standard patterns JCPDS file no. 47-1320 and JCPDS file no. 26-1273, respectively, indicating the high purity of the crystalline structure of the prepared oxide and oxygenated phosphide (Kumar et al., 2015; Liu et al., 2019). The A-MoO_x_ sample showed that dominant peaks at 12.8, 23.5, 25.7, 27.4, 33.7, and 49.3° correspond to the (001), (10**1̄**), (002), (011), (110), (102), and (020) crystal planes, respectively, while the dominant peaks of N-MoP located at 26, 37, and 53.5° correspond to the planes of (111), (131) and (**1̄**33), respectively. The morphological features of the developed products were studied using scanning electron microscopy (SEM) analysis. It is noticed that the sphere-shaped structures of the oxide as displayed in Figure 1B do not change even after the conversion to form phosphide as shown in Figure 1C. Further, high-resolution transmission electron microscope (HRTEM) results in Figures 1D,E delineate the lattice spacing of 0.344 and 0.272 nm for the (002) plane of crystalline A-MoO_x_ and N-MoP, respectively, matching well with the XRD findings. The elemental analysis was carried out to investigate the distribution of elements in the product. The EDS mapping of MoO_x_ clearly shows the homogenous dispersion of Mo and O in the product (Figures 1F,G) while N-MoP (Figures 1H,I and Figures S1, S2) indicates the homogeneous dispersion of P in the product with Mo and O. N is not detected in the EDS which might be due to less amount of N.

**Figure 2:**
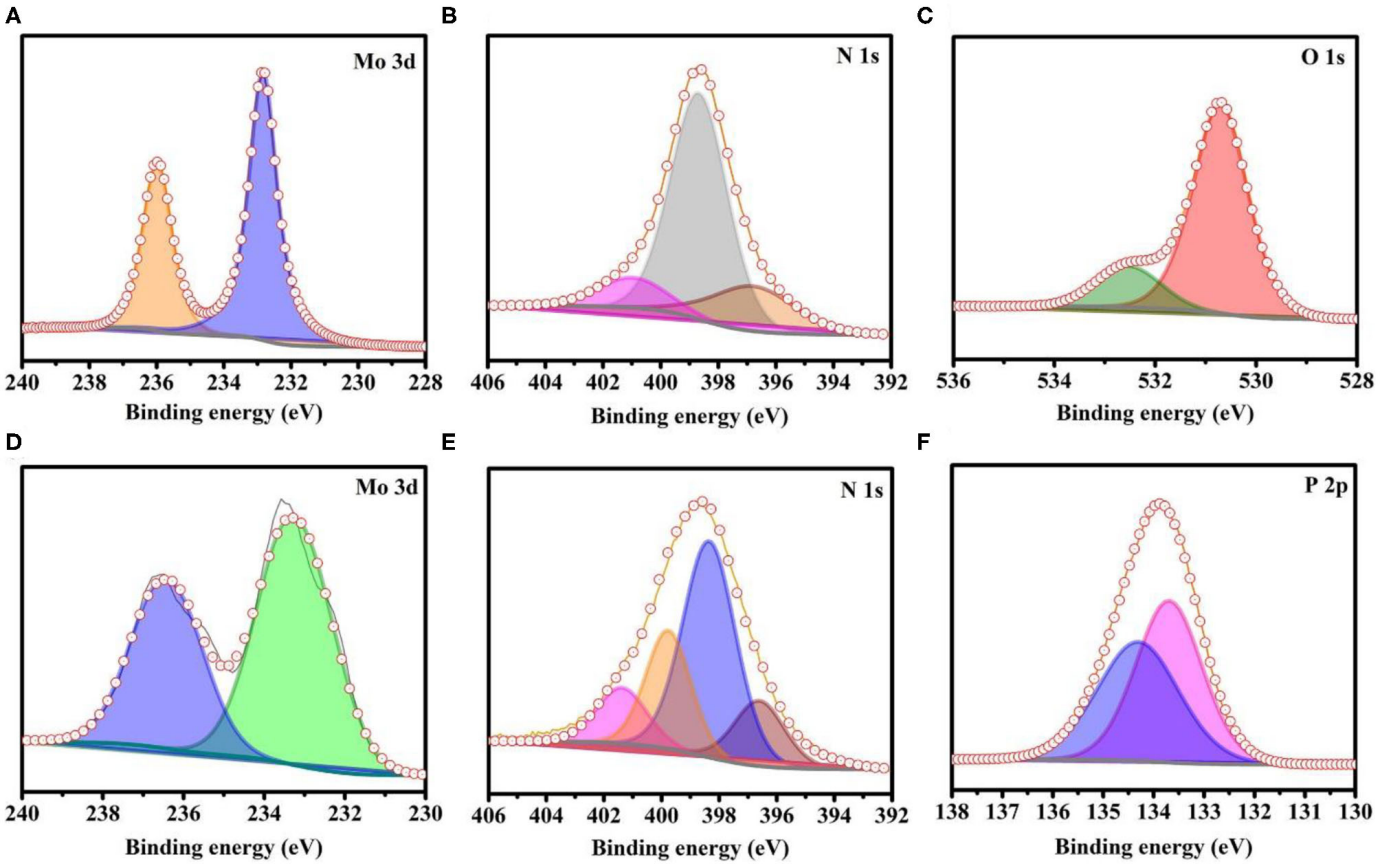
XPS analysis of as-synthesized samples; **(A)** Mo 3d, **(B)** N 1s, and **(C)** O 1s elements for annealed MoO_x_, **(D)** Mo 3d, **(E)** N 1s, and **(F)** P 2p elements for N-MoP.

X-ray photoelectron spectroscopy (XPS) was employed to investigate the presence of core levels of the elements as well as their oxidation states. The C1s peak (284.4 eV) was used as a reference to do the peak shift correction. The XPS results show the presence of Mo, N, and O (Figures 2A–C, respectively) in A-MoO_x_. The peak binding energy at ~236 and ~232.9 eV (Figure 2A) appears due to Mo 3d_3/2_ and 3d_5/2_, respectively, which points out the presence of the Mo–O bond (Li et al., 2015; Wen et al., 2019). The deconvoluted peaks of N1s appear in Figure 2B at ~396.7 and ~398.7 attributed to the N-doping in A-MoO_x_ and the peak at ~401 attributed to the surface-adsorbed N_2_ (Si et al., 2016; Haque et al., 2019). The peak at ~530.7 eV in Figure 2C can be assigned to the O–Mo bond and the other peak at ~532.7 eV to the adsorbed OH group and O–Mo bond (Luo et al., 2016; Zhang et al., 2018; Haque et al., 2019; Mohiuddin et al., 2020). It is evident from the results that Mo, N, and O are mainly present in A-MoO_x_, indicating the high purity of A-MoO_x_. The XPS analysis of N-MoP shows the presence of Mo, N, P, and O (Figures 2D–F and Figure S3) in the N-MoP sample. The doublet peaks of Mo 3d_3/2_ and 3d_5/2_ appear at ~236.7 and ~233.5 eV, respectively, in Figure 2D (Xing et al., 2014; Ren G. et al., 2019; Wen et al., 2019). The deconvolution peaks of N1s in Figure 2E at ~396.6, ~398.3, and ~399.8 represented the traces of doped N, and the peak at ~401 attributed to adsorbed N_2_ on the surface (Si et al., 2016; Haque et al., 2019). The deconvoluted peak at ~134.4 eV in Figure 2F suggests the presence of the P–O bond of phosphate (Xing et al., 2014; Mohiuddin et al., 2020), and the peak at ~133.7 eV shows the presence of the Mo–P bond, while the increase (or positively shift) in binding energy peak is attributed to the presence of a higher electronegative value of doped N in the structure (Mhtensson et al., 1995; Khattak et al., 1997; Blanchard et al., 2009; Zhang et al., 2014; Rai et al., 2018; Jeon et al., 2020) as higher electronegative N decreases the electron density to increase the binding energy and the molybdenum phosphate bond's peak centered around 133 eV (Khattak et al., 1997). The adsorbed OH groups on the surface of N-MoP show peaks at 531.9 and 533.4 eV of O1s, as shown in Figure S3. There is no peak that appeared around ~530.7 eV in N-MoP as present in the A-MoO_x_ sample attributed to the cleavage of the Mo–O bond during conversion and successful formation of the Mo–P bond as shown in Figure 2F after phosphorization. The results depicted that Mo, N, P, and O are mainly present in N-MoP attributed to the high purity of prepared N-MoP. Further, A-MoO_x_ having doped N and its conversion into oxygenated phosphide to form N-MoP are verified by XPS data. The presence of nitrogen and phosphorous species in N-MoP makes it a better electrocatalyst for HER than molybdenum oxides, owing to the enhanced conductivity (Wang D. et al., 2019; Wang L. et al., 2019).

**Figure 3:**
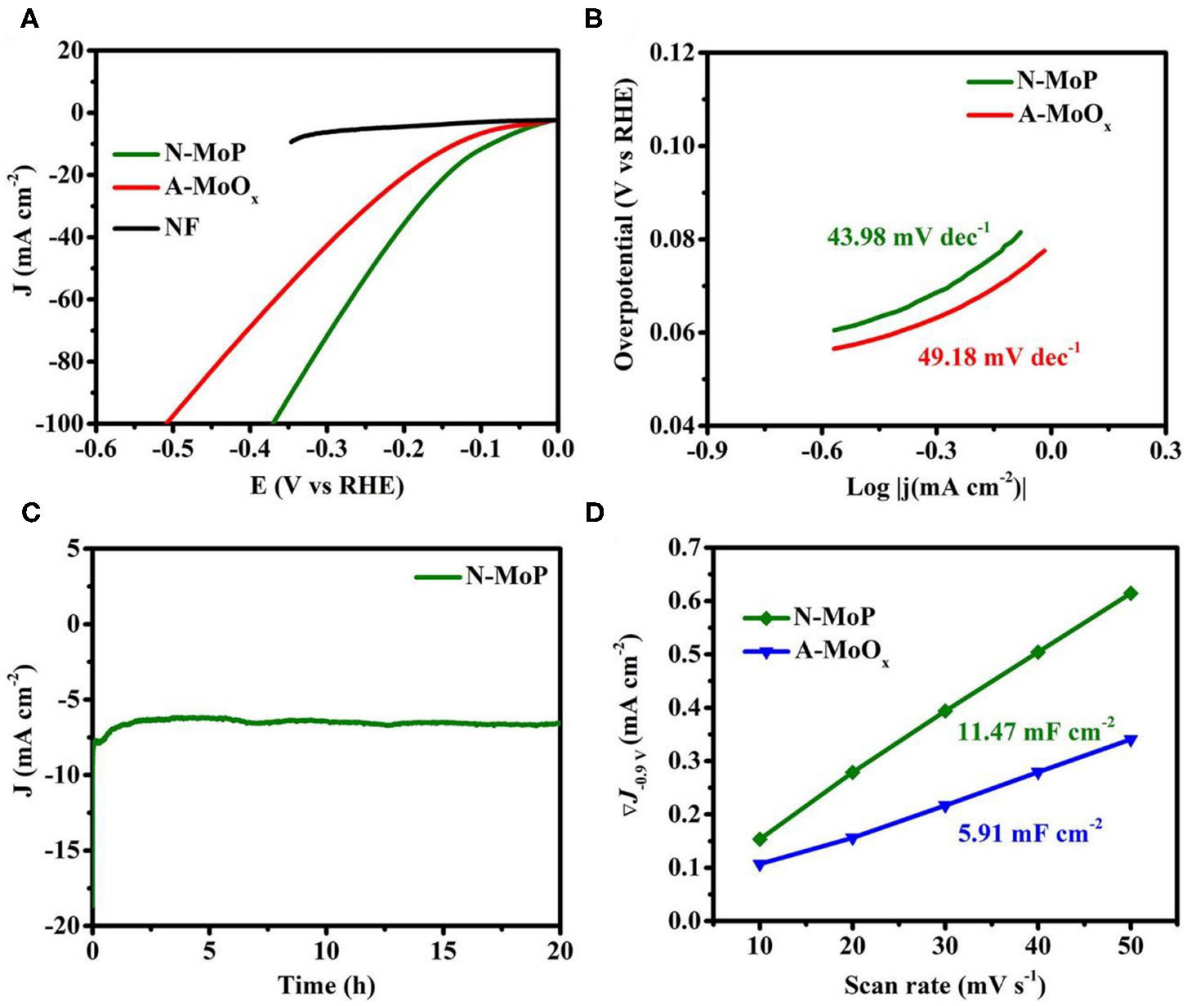
**(A)** Polarization curves for MoO_x_, annealed MoO_x_, N-MoP for HER, **(B)** Tafel plots, **(C)** the amperometric i–t plot for stability test of N-MoP, and **(D)** differences in current density (at a potential of 0.90 V) plotted against scan rate (the marked values in the graph are the fitted slopes) in 0.1 M KOH solution.

Inspired by the pure phase of N-MoP which assists fast mass transport, the electrochemical behavior of the as-developed N-MoP was investigated for the HER activity. The HER was carried out in 3-electrode configuration in 0.1 M KOH, and the results are presented in Figure 3. N-MoP exhibited excellent HER activity by reaching a current density of 10 mA cm^−2^ at a very low overpotential of only 87 mV, which is much superior to A-MoO_x_ 132 mV at 10 mA cm^−2^, as delineated by the polarization curves (Figure 3A), and the original reported MoO_x_ has 160 mV at 10 mA cm^−2^ (Haque et al., 2019). The higher activity can be attributed to the highly exposed surface, large electrode/electrolyte interface, and suitable binding energies for reactants which contribute toward improvement in overall catalytic performance. It can be assumed that the large channels for mass transport ensure fast reaction and evolution of H_2_, leading to exceptional performance. Furthermore, Mo acts as the hydride acceptor and P works as a proton acceptor. The presence of a large number of Mo–P bonds promotes the formulation of Mo-hydride, which facilitates the HER process on the developed catalyst (Xing et al., 2014). To further understand the reaction mechanism on the catalyst surface, the linear fitted Tafel slopes were plotted and are presented in Figure 3B. The Tafel values of 43.98 and 49.18 mV dec^−1^ were achieved for N-MoP and A-MoO_x_, respectively, suggesting that the reaction followed the Tafel–Heyrovsky mechanism (Figure 3B). The better values of Tafel slopes for N-MoP than oxide showed that N-MoP bears better reaction kinetics due to easy access of active sites and better desorption of hydrogen from the surface of catalysts.

Considering the industrial requirements, the long-term durability of N-MoP was measured by amperometric i–t curve technique, as shown in Figure 3C. The developed product shows the minimum loss in activity and current density for a period of 20 h. Such performance and stability assure that N-MoP can be used in commercial electrolyzers. Moreover, N-MoP exhibits much higher electrochemical double-layer capacitances of C_dl_ of 11.47 mF cm^−2^ compared to A-MoO_x_ (5.91 mF cm^−2^) (Figure 3D). Higher ECSA values assure a large electrode/electrolyte interface, more exposed active sites for the enhanced catalysis on the surface of the N-MoP, and higher conductivity of ions and electrons.

## Conclusions

The alternatives of precious noble metals have been studied to develop a new class of materials comprising phosphides of transition metals to produce clean energy through HER. N-MoP exhibited an overall particle-like sphere morphology with homogeneous dispersion of N and P in the product. N-MoP exhibited impressive HER activity in an alkaline environment by achieving a current density of 10 mA cm^−2^ at a very low overpotential of only 87 mV with a Tafel slope of 43.98 mV dec^−1^ as compared to A-MoO_x_ (132 mV at 10 mA cm^−2^ with Tafel slope of 49.18 mV dec^−1^). This enhanced performance is attributed to the extended Mo–P bond formation with assistance of N-doping, which facilitates the HER process. N-MoP also exhibits the long-term stability at least for 20 h with enhanced ECSA, indicating the more electrode/electrolyte interface, higher exposed active sites for the improved catalysis on the surface, and better conductivity of ions and electrons. This facile and simple synthesis of N-MoP with splendid HER performance will offer new possibilities for nitrogen-doped transition metal-based phosphides as an alternative to exorbitant noble metals.

## Data Availability Statement

The raw data supporting the conclusions of this article will be made available by the authors, without undue reservation.

## Author Contributions

All authors contributed in conducting experiments and writing manuscript.

## Conflict of Interest

The authors declare that the research was conducted in the absence of any commercial or financial relationships that could be construed as a potential conflict of interest.
