# Supplementary material for: Nitrogen-Doped Oxygenated Molybdenum Phosphide as an Efficient Electrocatalyst for Hydrogen Evolution in Alkaline Media
Source: Front Chem. 2020 Aug 28;8:733. doi: 10.3389/fchem.2020.00733 (PMC7484372; doi:10.3389/fchem.2020.00733)
Supplement: Supplementary file 1 [file Image_1.pdf]

Supplementary Information

Nitrogen-doped Oxygenated Molybdenum Phosphide as an Efficient Electrocatalyst for Hydrogen Evolution in Alkaline Media

Muhammad Waqas Khan,[a, b, 1], *Suraj Loomba,*[a, 1], *Rashad Ali,*[c] *Md Mohiuddin,*[a] *Ahmed Alluqmani,*[a] *Farjana Haque,*[a] *Yongkun Liu,*[d] *Rizwan Ur Rehman Sagar,*[e] *Ali Zavabeti,* [a, f] *Turki Alkathiri,*[a, g] *Babar Shabbir,*[h] *Jian Xian,*[c, i] *Jian Zhen Ou,* [a,]* *Asif Mahmood,* [j,] * *Nasir Mahmood* [a,]*

[a] School of Engineering, RMIT University, 124 La Trobe Street, Melbourne, Victoria, 3001, Australia.

[b] Applied Porous Materials Unit, Commonwealth Scientific and Industrial Research Organisation (CSIRO), Clayton, VIC 3168, Australia

[c] School of Materials and Energy, University of Electronic Science and Technology of China, Chengdu, 611731, China

[d] College of Textile Science and Engineering (International Institute of Silk), Zhejiang Sci-Tech University, Hangzhou, 310018, PR China

[e] School of Materials Science and Engineering, Jiangxi University of Science and Technology, Jiangxi, 341000, PR China

[f] Department of Chemical Engineering, The University of Melbourne, Parkville, Victoria 3010, Australia

[g] School of Engineering, Albaha University, Albaha, Alaqiq 4781, Saudi Arabia

[h] Department of Materials Science and Engineering, ARC Centre of Excellence in Exciton Science, Monash University, Clayton VIC 3800, Australia.

[i] National Engineering Researching Centre of Electromagnetic Radiation Control Materials, State Key Laboratory of Electronic Thin Films and Integrated Devices, University of Electronic Science and Technology of China, Chengdu, 611731, China

[j] School of Chemical and Biomolecular Engineering, The University of Sydney, Darlington, Sydney, New South Wales, 2006, Australia

Corresponding Authors

Dr. Jianzhen Ou: *jianzhen.ou@rmit.edu.au*

Dr. Asif Mahmood: *asif.mahmood@sydney.edu.au*

Dr. Nasir Mahmood: *nasir.mahmood@rmit.edu.au*

[1]These two authors contributed equally to this article.

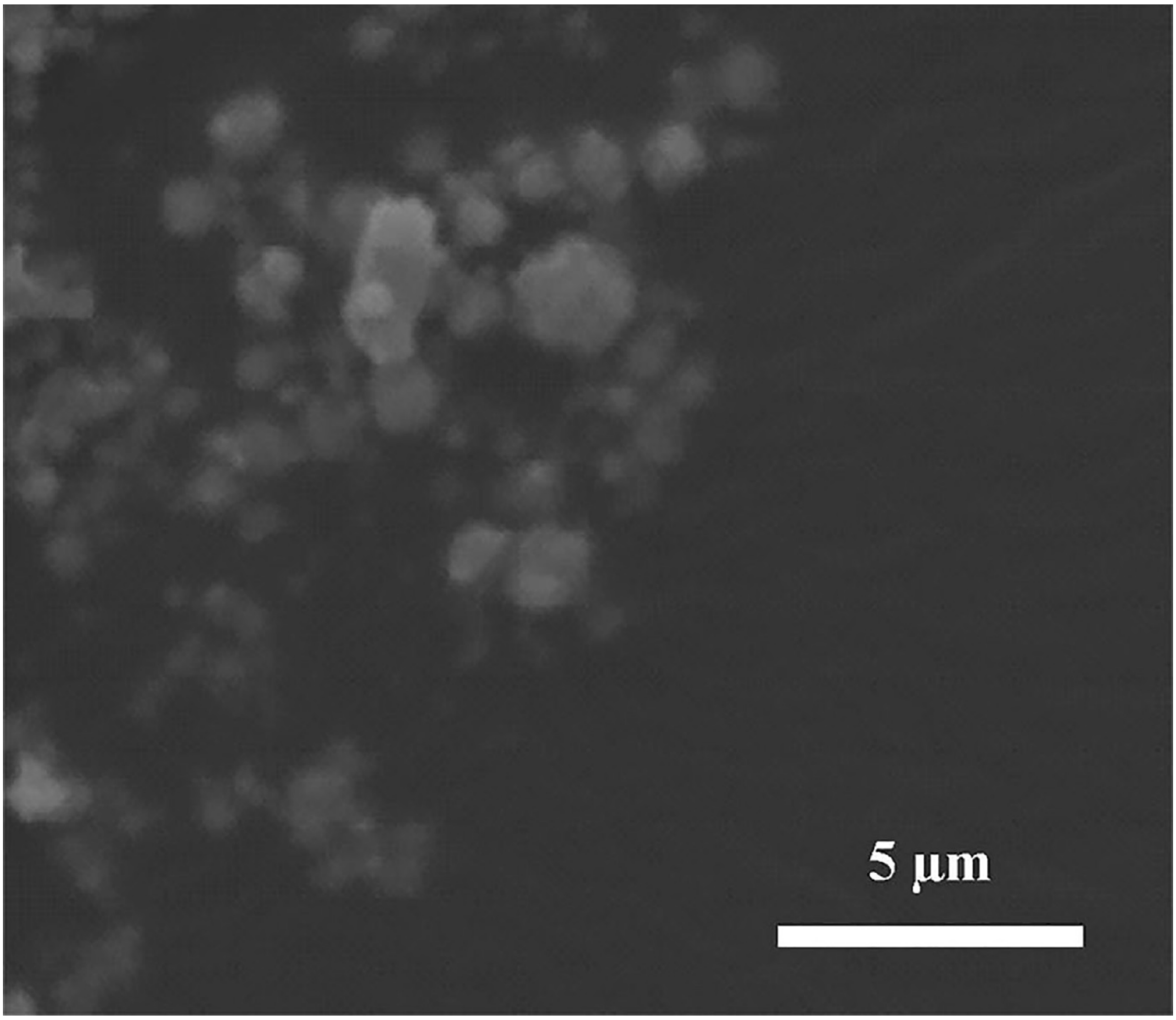

Figure S1: SEM micrograph of the N-MoP

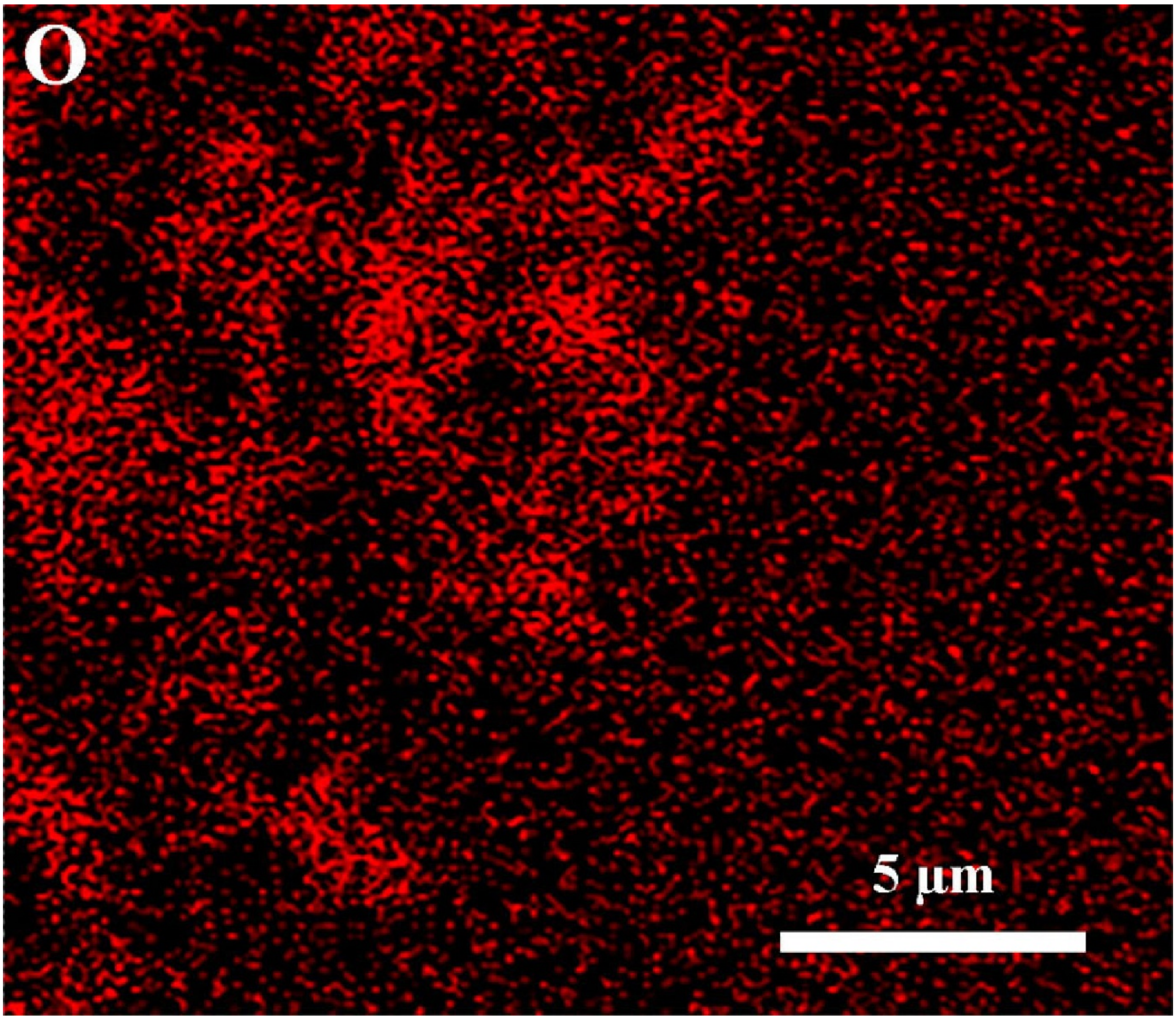

Figure S2: EDS mapping of O in the N-MoP

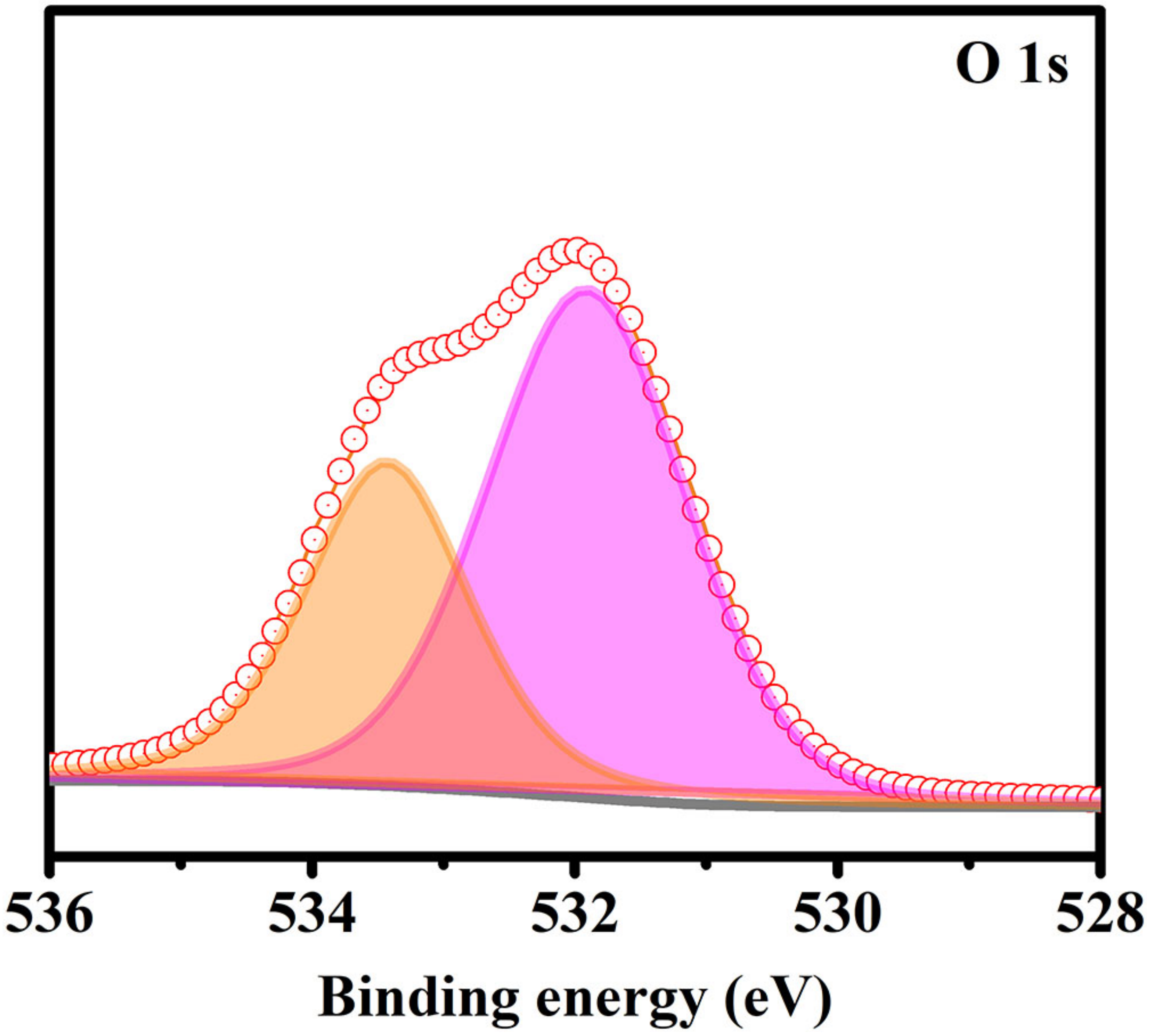

Figure S3: XPS spectra of O 1s for N-MoP
